# Diverse Immune Effects of Bovine Colostrum and Benefits in Human Health and Disease

**DOI:** 10.3390/nu13113798

**Published:** 2021-10-26

**Authors:** Subrata Ghosh, Marietta Iacucci

**Affiliations:** 1 APC Microbiome Ireland, College of Medicine and Health, University College Cork, T12 K8AF Cork, Ireland; 2 Institute of Immunology and Immunotherapy, University of Birmingham, Birmingham B15 2TT, UK; m.iacucci@bham.ac.uk

**Keywords:** immune effects, bovine colostrum, infections

## Abstract

The health benefits of bovine colostrum have extensively been studied, including immune effects mediated by immunoglobulins, lactoferrin, and casein, as well as by certain growth factors. Some of these effects are not directly related to the absorption of proteins from the intestinal tract. The ingestion of BC can modulate the function of subsets of lymphocytes, macrophages, and dendritic cells and increase regulatory cytokines such as interleukin 10. In this review, we predominantly focused on evidence from human studies on benefits in health and disease. This review highlights that clear evidence of the prevention of infectious diseases in pre-term infants such as necrotizing enterocolitis, neonatal sepsis or prevention of cancer metastasis is lacking. This is clearly an area where translational science has to be strengthened, taking the considerable evidence from numerous ex vivo studies on cells and tissues and from animal interventions. The review focuses predominantly on human data.

## 1. Introduction

Bovine colostrum (BC), generally produced by the milk industry, is composed of a number of immune-active proteins such as immunoglobulins, lactoferrin and other antimicrobial peptides, and casein, and is marketed commercially with claims to promote general health and immune support. BC may also be considered for the specific treatment of medical conditions in both children and adults. Commercially marketed colostrum may contain other constituents in addition to immune active molecules. The introductory article by Playford and Weiser [1] succinctly summarizes the overall composition as well as immune active micro- and macronutrients in BC and potential applications in health and disease, including performance and sports medicine, the prevention of infections in adults and infants, and in immune-mediated inflammatory diseases.

In large animal models, multicomponent nutraceutical supplementation containing BC has clearly been demonstrated to have beneficial effects on the host immune system and gut microbiota. A recent study intervention used the supplementary feeding of pigs using a multicomponent nutraceutical immunomodulatory and prebiotic cocktail composed of lactic acid bacteria, BC, dehydrated apple pomace, and essential oils [2]. This multicomponent feeding resulted in a statistically significant decrease in the proportions of T cytotoxic and double-positive (CD4+CD8+low) cells within the CD3+ cell population.

Conversely, a statistically significant increase in the proportions of B cells, and an increases IgG concentration and macrophage/monocyte cells compared to the beginning of the experiments was observed. Furthermore, significant changes in the bacterial composition of gut microbiota in pigs fed with multicomponent nutraceuticals were reported, with a higher number of probiotic strains such as Bifidobacterium, Streptococcus, and Faecalibacterium at the end of the experiment, compared to control group animals [2]. Such multi-component nutraceuticals should be further studied in human subjects.

**Figure 1:**
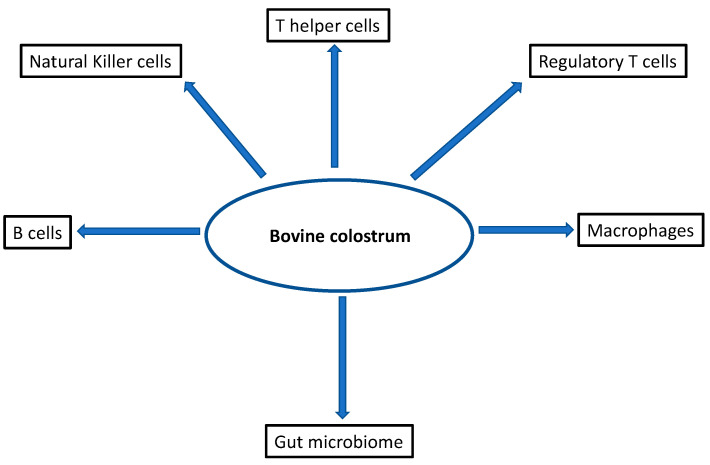
Immune cells that are affected by bovine colostrum.

Demonstrating clear and robust effects of BC in human subjects in health and disease has proven to be more challenging. In this review. we present evidence of the diverse immune effects of BC and potential resulting benefits in various health and disease conditions after the use of BC either on its own or as a component of multiple nutraceuticals (Figure 1). It can, however, be difficult to be certain of specific effects of BC that are mediated through immune modulation or through beneficial effects due to other mechanisms of action, such as intestinal permeability and regenerative effects.

## 2. Immune Effects of Colostrum in Health

Many of the effects of colostrum, especially BC in healthy adults, have been explored in the context of sports nutrition. These include immune effects and influences on intestinal permeability, as has been reviewed in detail by Davison in this series [3]. In athletes, upper respiratory tract infections are common and may be indicative of impaired immune responses related to prolonged heavy training and exertion. Changes in the phagocytic activity of blood neutrophils after the consumption of low-molecular-weight fractions from colostrum compared with placebos have been reported [4]. BC supplementation has been demonstrated to increase secretory IgA in saliva after 2 and 12 weeks of supplementation [5,6]. However, not all studies have been able to detect changes in secretory IgA concentrations after colostrum supplementation [7]. Davison has provided a comprehensive description and analysis, and sometimes conflicting evidence, in his article on colostrum supplementation in sports medicine [3].

However, a recent useful systematic review and meta-analysis specifically analyzed the immunological outcomes of BC supplementation in trained and physically active people, and reported that BC supplementation has no or a fairly low impact on improving the concentration of serum immunoglobulins (IgA and IgG), lymphocytes and neutrophils, and salivary immunoglobulin (IgA) [8]. This apparent lack of efficacy is even more stark given that the doses used in the included studies were 10–25 g/day, whereas commercial suppliers often advise a daily recommended dose of between 500 mg and 1 g/day. Systematic reviews are challenging due to heterogeneity of the studies, and further well-designed randomized studies are required before reaching a clear consensus on the value of BC supplementation in sports medicine and health to improve immune function. Of relevance is a recent randomized controlled trial in young female basketball players to investigate the effects of long-term BC supplementation on the immune system [9]. In this study, elite female basketball players either received BC or placebo orally twice daily for 24 weeks. A statistically significant change in plasma interleukin 10 levels in response to exercise program during supplementation period was observed in both groups, but other immune parameters such as interleukin (IL)-1alpha, IL-2, IL-13, TNF-alpha, insulin-like growth factor (IGF)-1, creatinine kinase (CK MM), immunoglobulin G (IgG), white blood cells, lymphocytes, monocytes, and granulocyte counts did not change and showed no effect of BC supplementation. However, whether measuring serum cytokine levels is a robust indicator of subtle immune boosting effects is debatable [9].

## 3. Colostrum for Immune-Nutrition in Elderly Subjects

Aging is associated with decreased immune responses, including the T lymphocyte immune response. This impairment may predispose aging population to infections, cancers and autoimmune diseases [10]. In a study on elderly, sedentary, non-smoking patients who met the inclusion criteria for immunogerontological studies and had low natural killer (NK) cell activity, a symbiotic mixture of *Saccharomyces boulardii* lysate in cranberry, colostrum-derived lactoferrin (which is bio-active for microbes), Fragaria, and lactose was administered twice daily for 2 months and compared with an identically tasting fruit juice placebo. A significant enhancement in NK cell activity of symbiotic mixture consumers was observed as compared to a placebo at the end of 2 months [11]. Of course, it is difficult to ascertain to what extent colostrum-derived lactoferrin was responsible for the effect on NK cell activity and whether this influenced protection against infections.

## 4. Immune Effects of Colostrum in Neonates and Babies

There is extensive research evidence of immune benefits in support of breast milk for newborns and infants. Human milk during the early lactation period can transfer key immune cells and proteins to the infants. These include macrophages and dendritic cells, which protect against pathogens [12], cytokines and growth factors, microRNAs, and mammary stem cells [13]. A recent exploratory study reported that the nature of nutrition supplements, BC, or human donor milk just after birth may affect gut microbiome development and nutrient metabolism in the neonatal period of preterm infants. BC-supplemented infants exhibited a lower relative abundance of the families *Lactobacillaceae* and *Enterococcaceae* than donor milk infants. The long-term implications of BC in pre-term infants require further studies [14].

Necrotizing enterocolitis (NEC) and neonatal sepsis are significant challenges in pre-term formula-fed infants. In a recent prospective double-blind controlled trial, 80 pre-term infants were randomly assigned to either a BC group (*n* = 32) or control group (*n* = 48) [15]. T lymphocytes and their subsets, necrotizing enterocolitis, late-onset sepsis (LOS) and its severity, feeding tolerance, growth, length of hospital stay, and mortality were documented. The BC group exhibited higher follow-up levels of CD4+CD25+ FoxP3+ T lymphocytes. Changes in FoxP3 Tregs levels between baseline and follow-up were mostly related to BC administration. The BC group also exhibited positive trends in the reduction in sepsis severity and mortality, with no significant differences in the incidence of NEC, LOS, or the length of hospital stay. However, the study was underpowered to establish the definite reduction in sepsis severity or concomitant mortality. Despite the influence on FoxP3 T regulatory cells, the study did not show significant effects on the incidence of NEC. A previous randomized trial also did not find evidence of the clinical efficacy of BC in the prevention of NEC, but this study too may well have been underpowered. However, in this study, if anything, there was a trend towards higher IL-6 and radiological features of NEC in the BC-treated group [16].

## 5. Use of Colostrum in Infectious Diseases

The beneficial effects of breast milk in reducing gastrointestinal infections in infants have raised the possibility of using BC to prevent or treat infections, especially affecting the intestinal tract. Polyvalent BC concentrates may inhibit intestinal LPS absorption in surgical patients, reducing peripheral blood LPS levels as well as IL-6 and CRP levels [17]. More studies are required to establish whether this can be clearly beneficial in the pre-operative setting. Lactoferrin in BC can induce the secretion of anti-inflammatory cytokines and T helper lymphocyte polarization, potentially preventing enteric infections and sepsis [18,19,20]. Environmental enteropathy associated with polymicrobial intestinal infections causes considerable morbidity and lowered response to oral vaccines in children in the developing world. Epithelial barrier damage induced by pathogens such as enteropathogenic *Escherichia coli* (EPEC), *Citrobacter rodentium* (*C. rodentium*), and *Cryptosporidium parvum* (*C. parvum*) reduces transepithelial resistance, whereas *C. rodentium* and *C. parvum* reduce claudin-4 expression and enable bacterial translocation through the epithelial monolayer. Enteropathogen-induced changes such as transepithelial resistance were improved by contra-pathogenicity agents, including BC [21,22].

## 6. Immune Effects of Colostrum on Colorectal Cancer

There is considerable interest in the inflammatory micro- and macro-environments in primary and secondary metastatic tumors in patients with colorectal cancer (CRC). The tumor microenvironment supports the migration process of tumor cells and their seeding in distant organs such as the liver and the lungs. Orally administered colostrum polyvalent immunoglobulins (KMP01D) increased interleukin (IL)-10 and IL-13 anti-inflammatory cytokine expression in patient-derived peripheral blood mononuclear cells (PBMCs) [23]. Interestingly, KMP01D also decreased the secretion of IL-1β, IL-6, interferon (IFN)-γ, tumor necrosis factor (TNF)-α, IL-12 inflammatory cytokines, and IGF-1 in these cells [23]. Moreover, CD14 and TLR4 expression involved in endotoxin signaling was downregulated in PBMCs and tumor-derived cells. Apoptosis of immune cells and tumor-derived cells was likewise enhanced with KMP01D. The addition of vitamin D3 as a cofactor demonstrated further enhancements of anti-inflammatory effects. In addition to oral administration, colostrum-derived KMP01D also demonstrated beneficial ex vivo effects on inflammatory cytokine responses in PBMCs and enhanced the apoptosis of immune cells from CRC patients. This study supported KMP01D as a treatment strategy to regulate stage-dependent local and systemic inflammation in CRC patients. However, evidence of the efficacy of BC on the immune tumor micro-environment is still too preliminary, although worthy of further exploration [20].

## 7. Conclusions

Beneficial immune effects of BC in health, especially in sports medicine, have been quite extensively explored. Whether such beneficial effects are mediated via the immune effects of BC is plausible, but not conclusively proven. Beneficial immune effects of BC have been demonstrated in animals such as pigs; however, in human subjects, clear biomarkers of an enhanced immune system as a defense against infections is lacking. Effects on T lymphocytes and natural killer cells have been demonstrated in vulnerable elderly populations, but more studies are required to establish clinical outcomes. In infants, enhanced regulatory T cells have been related to the administration of BC, but no clear effect on the prevention of NEC or sepsis could be confirmed. Changes in the gut microbiome after the administration of BC and lactoferrin mediated anti-microbial and immune effects represent significant potential in preventing infections; such effects may be most beneficial in undernourished children in the developing world. Whether modifications of the gut microbiome and modulation of the tumor micro-environment in cancers, especially colorectal cancer, can be beneficial in preventing metastasis is an intriguing concept. More translational studies are clearly required, taking into account the variable bioactivity of commercially produced BC. Evidence-based systematic reviews are often hampered by heterogenous and poor-quality studies [24].

## Data Availability

Not applicable.
